# The effect of parenteral selenium on outcomes of mechanically ventilated patients following sepsis: a prospective randomized clinical trial

**DOI:** 10.1186/s13613-015-0071-y

**Published:** 2015-10-01

**Authors:** Legese Chelkeba, Arezoo Ahmadi, Mohammad Abdollahi, Atabak Najafi, Mohammad Hosein Ghadimi, Reza Mosaed, Mojtaba Mojtahedzadeh

**Affiliations:** Department of Clinical Pharmacy, Faculty of Pharmacy, Tehran University of Medical Sciences, International campus (TUMS-IC), Tehran, Iran; Department of Anesthesiology and Critical Care Medicine, Faculty of Medicine, Sina Hospital, Tehran University of Medical Sciences, Tehran, Iran; Faculty of Pharmacy and Pharmaceutical Sciences Research Center, Tehran University of Medical Sciences, Tehran, Iran; Department of Clinical Pharmacy, Colleague of Public Health and Medical Sciences, Jimma University, Jimma, Ethiopia

**Keywords:** Severe sepsis, Septic shock, Selenium, Ventilator-associated pneumonia

## Abstract

**Background:**

Sepsis and septic shock is characterized by oxidative stress that mainly promotes systemic inflammation and organ failure due to excessive free radical production and depletion of antioxidant defenses. Therefore, we investigated the effect of selenium administration on antioxidant status, levels of cytokines and clinical outcomes.

**Methodology:**

This study was a prospective randomized control trial (RCT) whereby patients received selenium as sodium selenite (2 mg IV bolus followed by 1.5 mg continuous infusion for 14 days) plus standard therapy. The control group received standard therapy without selenium. The primary endpoint was 28-day mortality. The changes in the mean levels of glutathione peroxidase (GPX) activity, IL-6, IL-8 and IL-10, the incidence of ventilator-associated pneumonia (VAP) and other secondary endpoints were also recorded. VAP was broken down into early VAP and late VAP to see the clinical significance of each. We also recorded any adverse outcomes from selenium infusion.

**Results:**

Over 24-month period, 54 patients were recruited and randomized and an intention to treat (ITT) principle was applied (selenium, *n* = 29; control, *n* = 25) in the final analysis. There was no statistically significant difference between the two groups in 28-day mortality although it was lower in the selenium group compared with the control group: 9 (31 %) in the selenium versus 10 (40 %) in the control groups (*p* = 0.49). At day 0, GPX activity was 0.185 ± 0.3 versus 0.19 ± 0.3 U/mL (*p* = 0.9), day 3, GPX activity was 0.52 ± 0.5 versus 0.17 ± 0.2 U/mL (*p* = 0.02), at day 7 it was 0.55 ± 0.5 versus 0.24 ± 0.3 U/mL (*p* = 0.032), at day 10 it was 0.62 ± 0.7 versus 0.33 ± 0.4 U/mL (*p* = 0.048) and at day 14 it was 1.1 ± 1 versus 0.89 ± 1 U/mL (*p* = 0.70) for the selenium versus control groups, respectively. However, there were no significant differences between the mean plasma levels of all the three inflammatory cytokines at any point in time between the two groups. There was a significant reduction in occurrence of VAP in the selenium group compared with the control group (55.2 versus 84 %, *p* = 0.023), respectively.

**Conclusion:**

High-dose selenium administration within the time frame of early goal-directed therapy was not resulted in reduction of 28-day mortality, but increased the activity of glutathione peroxidase with no effect on the levels of inflammatory cytokines at any point in time in mechanically ventilated septic patients. However, selenium supplementation in mechanically ventilated patients following sepsis was associated with reduced occurrence of VAP.

Trial registration: IRCT201212082887N4 at WHO Clinical Trial Registry, August 29, 2014

## Background

Sepsis constitutes a major health care problem. The incidence of severe sepsis has increased over time and the projected estimate is increased by 1.5 % ever year due to number of reasons [1–3]. Population data suggested that severe sepsis is the leading cause of hospitalization and accounted for 2 % of all hospital admission, with 59 % of the patients requiring admission to the intensive care unit (ICU) [4]. Although the mortality of severe sepsis and septic shock decreased over time, it is still remaining unacceptably high [3, 5].

A number of studies suggested that inflammatory cytokines and oxidative stress released during sepsis were higher in septic patients and their concentrations were associated with severity and evolution of organ dysfunctions and death [6–9]. In the initial phase of the disease, there is an overwhelming inflammation, whereby migration of the neutrophils into the inflamed tissue releases free radicals that damage the endothelium and epithelium tissue leading to capillary congestion, leukocyte and macrophage infiltration into the site of inflammation of the respiratory system leading to respiratory failure [10]. Consequently, they acquire nosocomial respiratory tract infections after 48 h of mechanical ventilation; ventilator-associated pneumonia (VAP) [11]. It has been reported that ventilator-associated pneumonia is affecting more than 30 % of those susceptible patients and increasing morbidity, mortality, length of ICU stay and costs [12–15]. Hence, therapies counteracting the inflammation and oxidative stress and the consequences are attractive. One of those therapies seems to be selenium supplementation. Selenium is an essential trace element for the biosynthesis and function of about 25 known selenocysteine containing selenoproteins, located on the catalytic center of most selenoenzymes [16]. One of the best known and characterized redox systems is glutathione complex consisting of the selenium-dependent peroxidases and the thioredoxin reductases [17, 18]. Study suggested that selenium plays an important role as anti-inflammatory agent by tightly regulating the expression of proinflammatory genes in immune cells [19, 20].

Low plasma selenium levels are associated with an increasing risk of nosocomial infections [21]. Daily infusion of 1600 µg selenium following initial bolus dose of 2000 µg for 10 days was also found to be a novel therapy that increases Se status, improves illness severity, and lowers the incidence of hospital-acquired pneumonia for systemic inflammatory response syndrome (SIRS) patients in ICU [10]. However, randomized trials and their pooled data involving parenteral selenium supplementation in critically ill patients with sepsis have yielded contradictory results [22–26]. Currently, we do not have concrete evidences that support the administration of selenium in septic patients. Therefore, doing trials that incorporated biomarkers to optimize treatment effects could build the bodies of literatures and help clinicians that need evidence-based medicine. We hypothesized selenium administration within the time frame of early goal-directed therapy in mechanically ventilated patients following sepsis (1) reduces 28-day mortality by 20 % (2) reduces oxidative stress and inflammation (3) reduces ventilator-associated pneumonia.

## Methods

### Study design and patient selection

This was a prospective, randomized, single blind, single center clinical trial conducted on 54 septic patients admitted to ICU of Sina Hospital of Tehran University of Medical Sciences (TUMS). Consecutive patients were recruited from 2012 to December 2014. The study was conducted in accordance with the declaration of Helsinki and approved by the ethics committee of Tehran University of Medical sciences international campus (TUMS-IC) [code of ethical approval: 1-1: 90-3-29]. Oral and written informed consent was obtained from the patients or their close relatives. Moreover, an identification code was used instead of patient´s name to protect the patient´s identity when reporting trial-related data.

### Inclusion and exclusion criteria

We included patients of age ≥17 years with sepsis, severe sepsis and septic shock, enrolled into the study after diagnosis within 6 h, if the patient was on mechanical ventilation >48 h and if they had informed consent either from the patient or the relative. On the other hand, patients were excluded if they were of age <17 years, if they were pregnant, if they had missing informed consent either from the patient or the relative, if they participated before in this clinical trial, if they had cancer as the cause of SIRS or sepsis, chronic kidney diseases and if medical staff decided to limit care.

### Randomization and protocol

Consecutive eligible patients were recruited and randomized via block randomization into four blocks, in which a random selection was done using a list of numbers generated using statistical software. Half of the patients in each block were allocated to selenium and the remaining half to control group. Boxes containing the whole treatment for each patient were delivered to the investigator by the hospital pharmacist following the order of the randomization list. All patients remained blinded throughout the study period. Standard treatments for severe sepsis and septic shock were given according to the surviving sepsis campaign (SSC) [27] guidelines. Patients in selenium group were received 2000 µg of sodium selenite in 100 mL of normal saline within the first 6 h of diagnosis of sepsis during 1 h intravenously followed by 1500 µg of sodium selenite in 250 mL of normal saline during 12 h continuously for 14 days. The control groups received standard therapy without selenium. Patients otherwise were treated according to the best practice of the hospital, including parenteral or enteral nutrition together with vitamins and trace elements as necessary. The intervention drug was supplied by Biosyn Arzneimittel GmBH (Fellbach, Germany).

### Definitions

Sepsis, severe sepsis and septic shock were defined according to criteria proposed by the American College of Chest Physicians/Society of Critical Care Medicine [28]. Standard treatments for severe sepsis and septic shock were implemented as following: measurement of lactate levels, appropriate diagnosis studies to ascertain causative organisms before starting antibiotics, early administration of broad spectrum antibiotic therapy, early fluid administration to target Central Venous Pressure (CVP) of 8–12 mmHg and central venous oxygen saturation (SvO_2_) >70 %, hypotension control with vasopressor [27]. Additional works accomplished within 24 h as appropriate were low dose steroid, glycemic control (blood glucose <150 mg/dL), lung protective ventilation and standard prophylactic measures for deep vein thrombosis (DVT) and stress related mucosal damage (SRMD). We defined VAP as pneumonia that occurs 48–72 h or thereafter following endotracheal intubation, characterized by the presence of a new or progressive infiltrate, signs of systemic infection (fever, altered white blood cell count), changes in sputum characteristics, and quantitative detection of a causative agent; early VAP <5 days, late VAP ≥5 days of mechanical ventilation [29]. SOFA respiratory score was calculated based on PaO_2_/FiO_2_ (mmHg)—SOFA score <400, 1; <300, 2; <200 and mechanically ventilated, 3; < 100 and mechanically ventilated, 4.

### Data collection

#### Clinical data

We recorded the following data: demographic data, vital signs, severity scores by calculating APACHE II score, SOFA score, comorbidities, and type of infection based on culture results.

#### Laboratory data

We recorded data on hematological, biochemical data analysis, blood gas (for determination of (PaO_2_/FiO_2_), blood cultures and cultures of specimen drawn from the site of infection on randomization and then as appropriate.

#### Follow-up

Patients were followed up for 90 day or till they died, depending which happened first. The following variables were collected on days 3, 7, 10 and 14 after randomization: vital signs, SOFA respiratory scores and standard laboratory tests. Cultures of specimens drawn from any new site of infection were performed throughout the ICU stay. Duration of mechanical ventilation, duration of vasopressor support, duration of ICU and hospital stays, the incidences of new infection, incidence of ARDS, 28-day mortality and overall mortality were also recorded. The occurrence of VAP was noted throughout the ICU stay along with any adverse reaction as a result of selenium infusion.

### Outcome measures

The primary outcome was 28-day mortality. Changes in GPX activity, IL-6, IL-8 and IL-10 in different points in time of study, duration of mechanical ventilation, duration of ICU and hospital stays and incidence VAP. We also evaluated VAP as early VAP versus late VAP and the effect of each on some clinical outcomes and health care resource consumption measures.

### Sample collection, handling and analysis

Blood samples (5 mL each) were taken from central venous catheters and arterial lines. The first sample was obtained upon diagnosis of sepsis, but prior to initiation of the therapy. Other samples were obtained on days 3, 7, 10, and 14 after randomization. The blood samples were collected into vacutainer tubes containing EDTA and spun these samples at 3000×*g* for 10–15 min to remove cells and cellular debris. The cell-free supernatant and plasma were stored at −80 °C until the time of the analyses. The levels of GPX, IL-6, IL-8 and IL-10 were analyzed via automated platinum ELISA kit (Human Cu/ZnOD, Affymetrix eBioscience, Vienna, Austria) in Massoud laboratory, an independent laboratory found in Northern part of Tehran, Iran following the manufacturing’s instruction. All other routine laboratory activities were carried out in the hospital laboratory according to the prescription of the attending clinicians.

The primary end point was 28-day mortality in the selenium groups and control group. A sample size calculation was carried out using these parameters: alpha-level 0.05, statistical power level 0.8, anticipated effect size of 0.2. This resulted to a suggested sample size of 90 patients based on the results of previous study [30]. However, the recruitment process was so slow that we included only 54 patients in our final analysis according to ITT principle. Recruitment process was so slow due to the fact that Sina hospital is trauma center I which gives services for patients referred from other centers in the country. Since we are caring for much debilitated patients referred from other centers that require intensive monitoring and our restricted inclusion criteria, we could not get as many patients as we planned.

### Statistical analysis

Discrete variables are expressed as counts (percentage) and continuous variables as mean ± standard deviation (SD). Categorical data were compared using Fisher’s exact test or Pearson Chi-square test. A Kolmogorov–Smirnov test was used to verify the normality of distributions of continuous variables. Continuous variables conforming to a normal distribution were compared using Student’s *t* test otherwise the Mann–Whitney *U* test was applied. Changes in SOFA respiratory scores and plasma cytokines levels over time as a function of group were analyzed by performing the two-way repeated-measures of variance (ANOVA). *P* value <0.05 was considered as statistically significant.

## Results

### Patients’ selection and recruitment

**Fig. 1:**
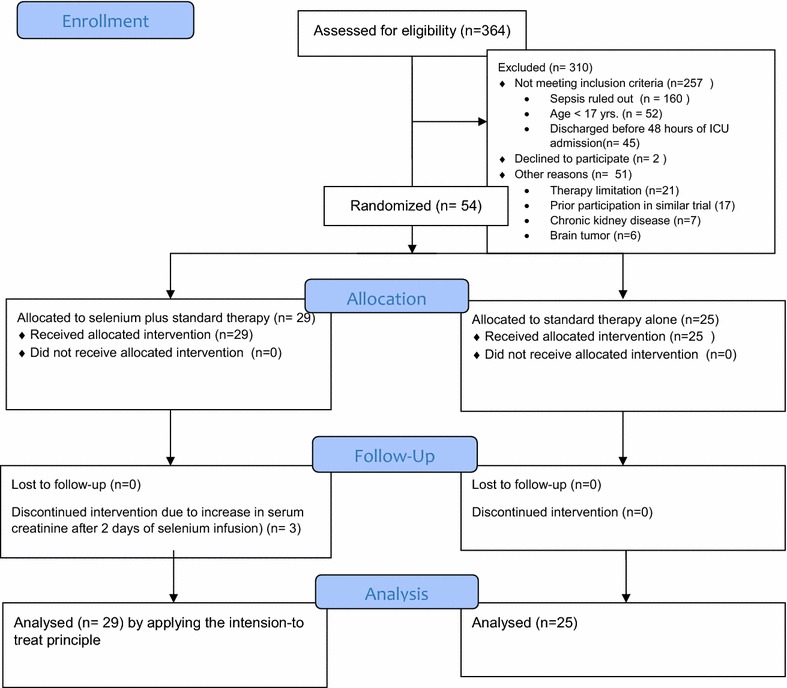
The profile of study design and patient selection processes

We assessed 364 patients for eligibility, of which 310 patients were excluded for the following reasons: 257 patients were excluded for not fulfilling the inclusion criteria, 2 patients decline not to participate and 51 patients for different reasons as described in detail in Fig. 1. Finally, 54 patients fulfilling the inclusion criteria recruited over a 2-year period (Selenium = 29, control = 25) were included in our final analysis according to the ITT principle although selenium was discounted in 3 patients due to rise in serum creatinine by the order of the senior clinician after 2 days of infusion. All patients followed either till they died or for 90 days, whichever happened first.

### Patient characteristics at randomization

There were no significant differences between the two patient groups for the general characteristics at randomization. The median (IQR) age of patients included was 35 (177–82) years for selenium group and it was 41 (19–82) years for the control group, of which 46 patients (85.2 %) were male. The mean SOFA, APACHE II score and PaO_2_/FiO_2_ on the day of admission were similar in both groups. The severity, the site of infection and the type of organism involved did not significantly differ between the two groups at baseline as shown in Table 1.

**Table 1:** Demographic and baseline characteristics of study groups

Characteristics	Selenium (*n* = 29)	Control (*n* = 25)	*P* value
Age	35 (17–82)	41 (19–82)	0.18^a^
Male/female^b^	24/5	22/3	0.59
APACHE II^b^	17 ± 4.3	16.4 ± 4.0	0.71
SOFA^b^	8 ± 2.7	8.3 ± 3	0.69
PaO_2_/FiO_2_^b^	160 ± 89	150 ± 75	0.65
Sepsis, *n* (%)	7 (24.1)	6 (24)	0.90
Severe sepsis, *n* (%)	8 (27.6)	7 (28)	0.89
Septic shock, *n* (%)	14 (48.3)	12 (48)	0.86
Pneumonia, *n* (%)	18 (63)	19 (76)	0.7
Peritonitis, *n* (%)	7 (24)	3 (12)	0.20
SSTI infection, *n* (%)	3 (10)	2 (8)	0.67
CNS infection, *n* (%)	0 (0)	1 (4)	0.33
CR infection, *n* (%)	1 (3)	0 (0)	0.32
Gram positive, *n* (%)	4 (13.7)	1 (4)	0.20
Gram negative, *n* (%)	8 (27.6)	9 (36)	0.9
No growth, *n* (%)	17 (58.7)	15 (60)	0.87

*APACHE II* acute physiological and chronic health evaluation II, *SOFA* sequential organ failure assessment, *SSTI* skin and soft tissue, *CNS* central nervous system, *CR* catheter line related

^a^Data given as media (IQR)

^b^Data given as mean ± SD

### Study endpoints

*28-day mortality* There was no statistically significant difference between the two groups in 28-day mortality although it was lower in the selenium group compared with the control group; 9 (31 %) in the selenium group compared with 10 (40 %) in the control group, (*p* = 0.49).

**Fig. 2:**
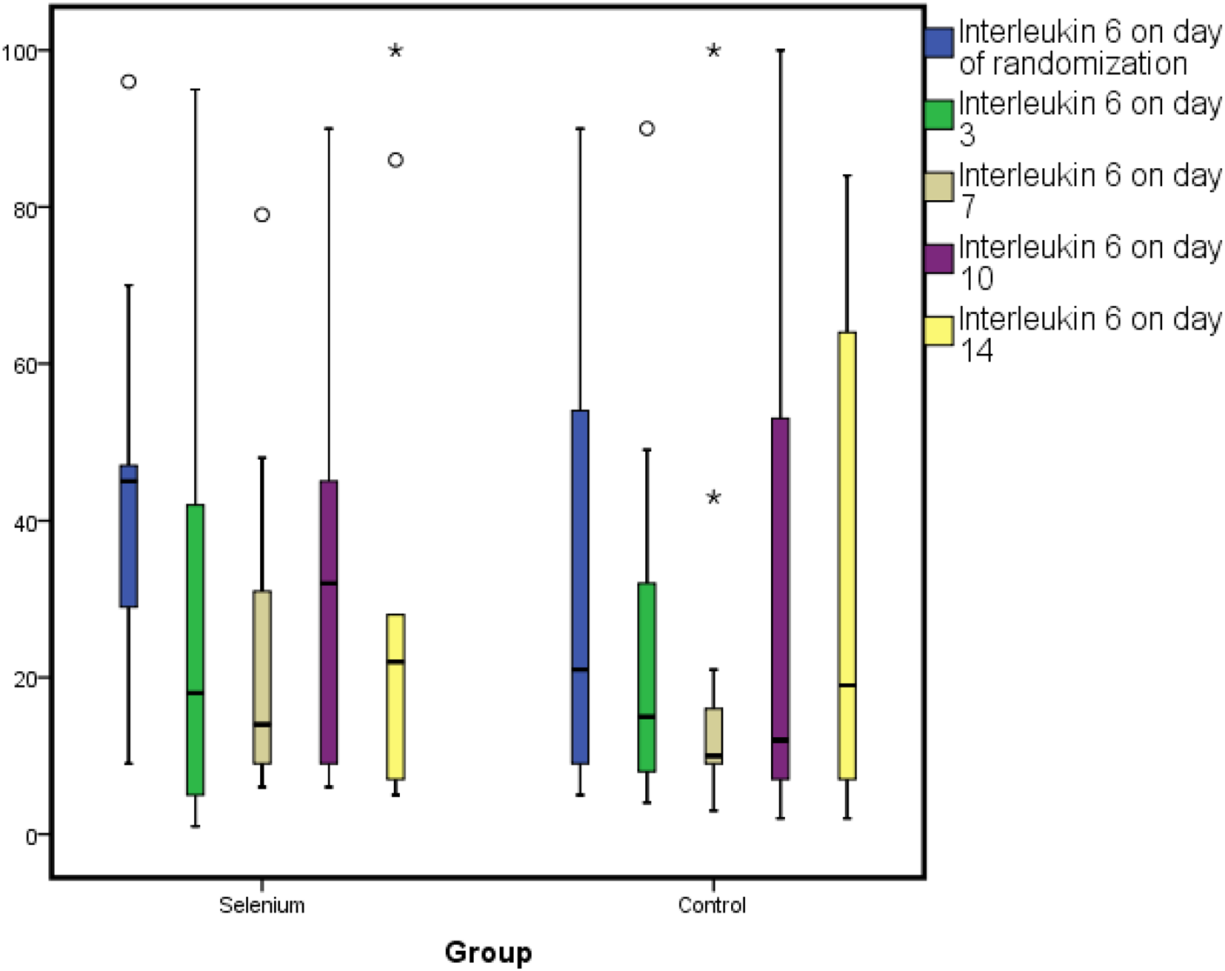
A box plot showing changes in IL-6 over time period of study patients. Figure shows mean ± SD

**Fig. 3:**
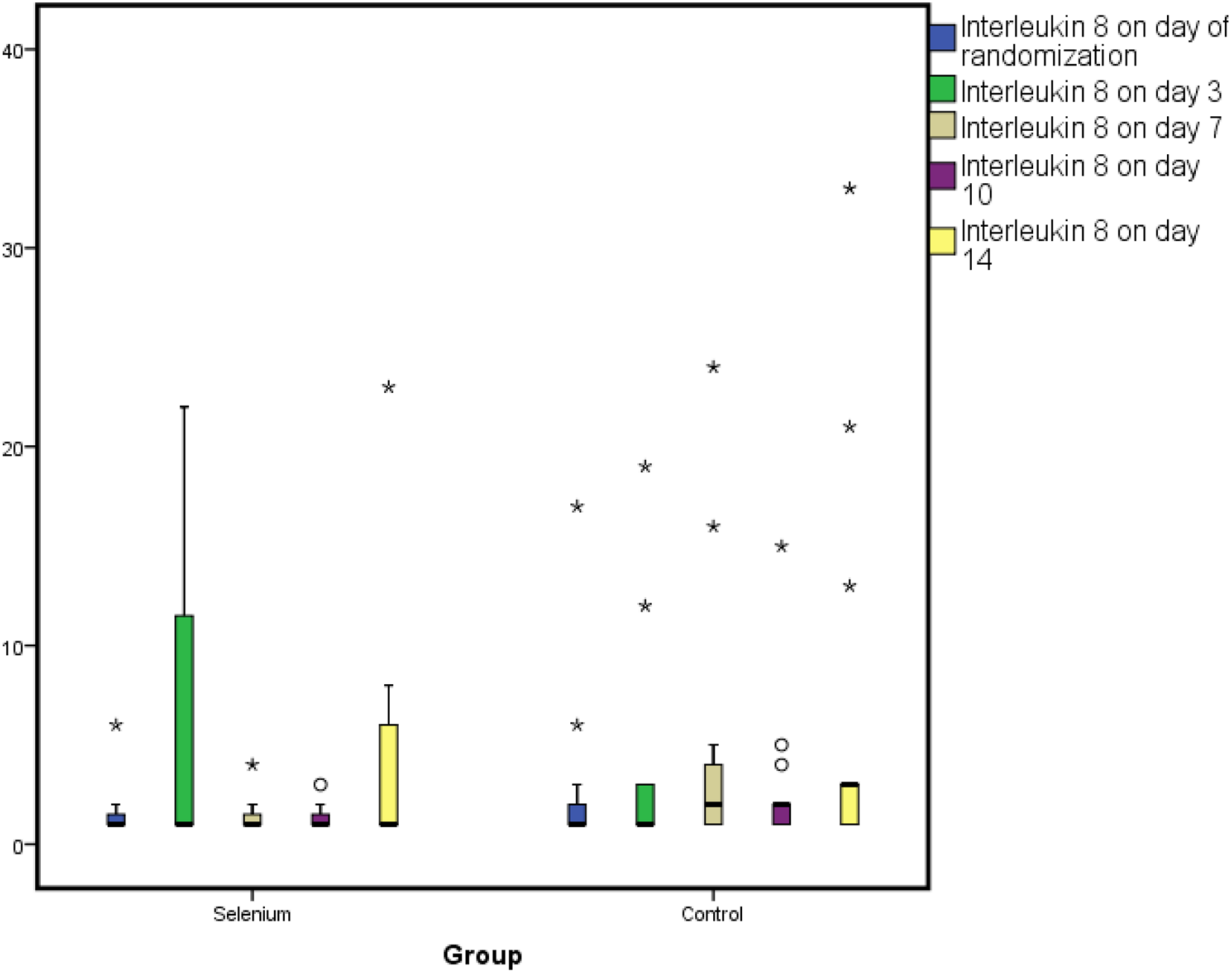
A box plot showing changes in IL-8 over time period of study patients. Figure shows mean ± SD

**Fig. 4:**
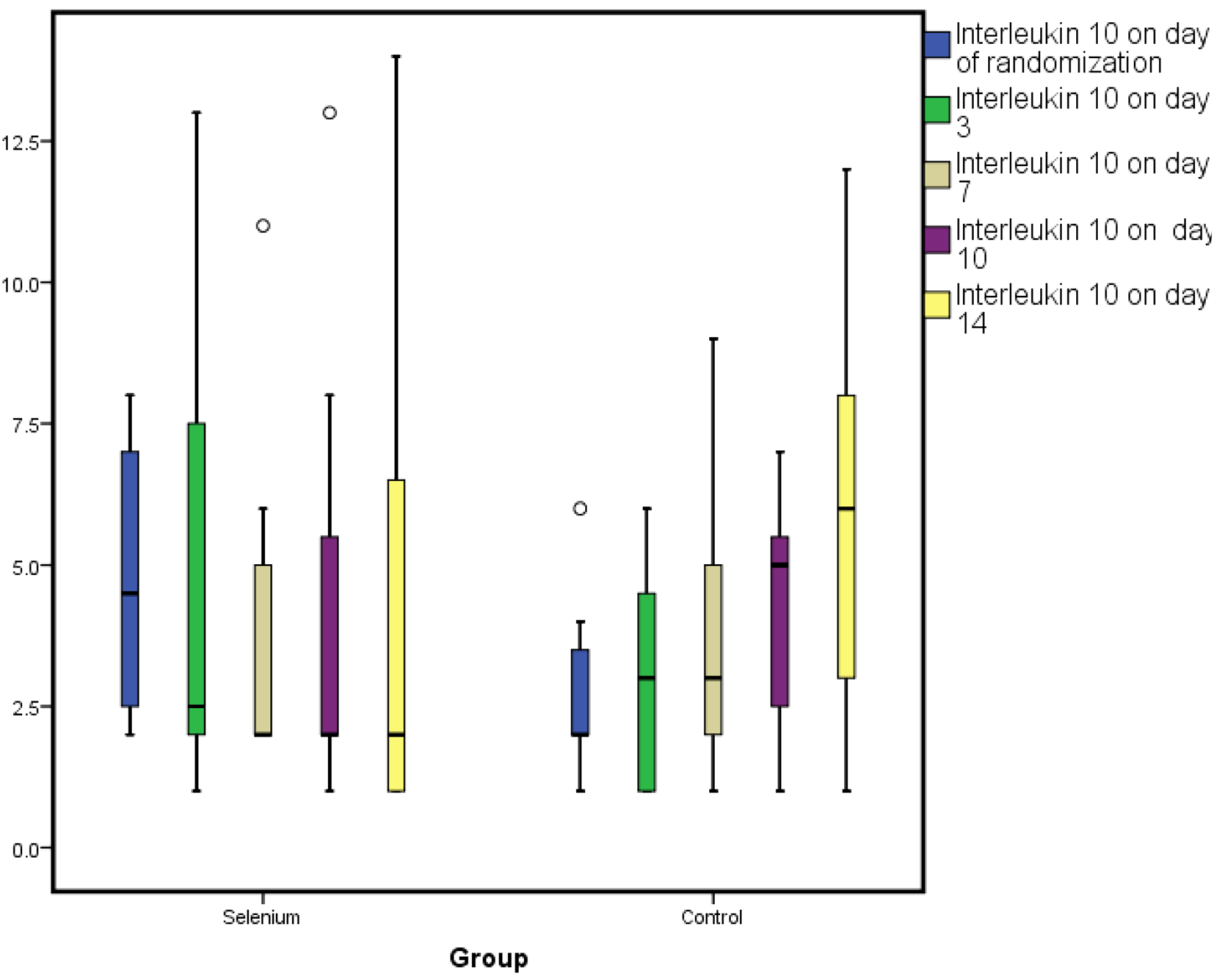
A box plot showing changes in IL-10 over time period of study patients. Figure shows mean ± SD

*Change in GPX, IL-6, IL-8 and IL-10* At day 0, GPX activity was 0.185 ± 0.3 versus 0.19 ± 0.3 U/mL (*p* = 0.9); day 3, GPX activity was 0.52 ± 0.5 versus 0.17 ± 0.2 U/mL (*p* = 0.02); at day 7, it was 0.55 ± 0.5 versus 0.24 ± 0.3 U/mL (*p* = 0.032); at day 10, it was 0.62 ± 0.7 versus 0.33 ± 0.4 U/mL (*p* = 0.048) and at day 14 it was 1.1 ± 1 versus 0.89 ± 1 U/mL (*p* = 0.70) for the selenium versus control groups, respectively. The GPX levels reached significant levels at day 3 and continued to rise till day 14. There were no significant differences between the mean plasma levels of all the three inflammatory cytokines at any point in time between the two groups as shown in Table 2 and Figs. 2, 3, 4.

**Table 2:** Changes in the levels of GPX, IL-6, IL-8 and IL-10 over time period in study patients

Biomarker (pg/mL)	Selenium group	Control group	*P* value
Glutathione peroxidase (U/mL)			
GPX on day 0	0.185 ± 0.3	0.19 ± 0.3	0.90
GPX on day 3	0.52 ± 0.5	0.17 ± 0.2	0.02
GPX on day 7	0.55 ± 0.5	0.24 ± 0.3	0.032
GPX on day 10	0.62 ± 0.2	0.33 ± 0.1	0.048
GPX on day 14	1.1 ± 1	0.89 ± 1	0.70
IL-6 (pg/mL)			
Day 0	43.3 ± 34	37.8 ± 35.6	0.61
Day 3	25 ± 28	32.7 ± 33.3	0.42
Day 7	21.6 ± 27.6	18.6 ± 24	0.75
Day 10	32.5 ± 31.8	29.8 ± 33.5	0.82
Day 14	30.9 ± 33.8	31.4 ± 30	0.97
IL-8 (pg/mL)			
Day 0	4.1 ± 5	4 ± 5.0	0.95
Day 3	5.7 ± 7.5	4.5 ± 6.8	0.58
Day 7	5.8 ± 8	5 ± 6.8.0	0.78
Day 10	4.8 ± 8	5.8 ± 5	0.73
Day 14	6.1 ± 8	6.1 ± 9	0.99
IL-10 (pg/mL)			
Day 0	4.6 ± 3	3.6 ± 2	0.26
Day 3	4.6 ± 3.7	4.2 ± 4.5	0.76
Day 7	3.8 ± 2.8	5.8 ± 5.7	0.21
Day 10	3.8 ± 3.5	6.2 ± 6	0.23
Day 14	3.9 ± 4.6	6 ± 4.6	0.26

Data presented in mean ± SD

*GPX* glutathione peroxidase, *IL-6* interleukin-6, *IL-8* interleukin-8, *IL-10* interleukin-10

**Fig. 5:**
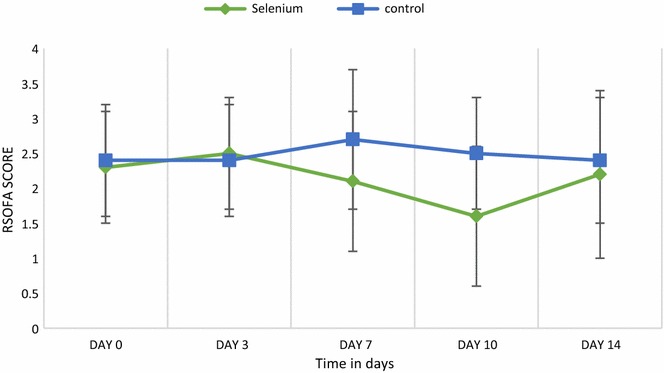
Changes in the mean SOFA respiratory scores in study patients over time period. Figure shows mean ± SD

*Clinical outcomes* The mean SOFA respiratory scores on the day of randomization (2.3 ± 0.8 versus 2.4 ± 0.8, *p* = 0.82) and day 3 (2.5 ± 0.9 versus 2.4 ± 0.9, *p* = 0.69) were similar in the selenium and control groups, respectively. However, the mean SOFA respiratory scores decreased significantly only in the selenite versus control group, respectively (2.1 ± 1 versus 2.7 ± 1, *p* = 0.03) at day 7 and (1.6 ± 1.2 versus 2.5 ± 0.8, *p* = 0.01) day 10 as shown in Fig. 5. VAP was diagnosed in 16 (55.2 %) patient in the selenite group compared with 21 (84 %) patients in the control group, (*p* = 0.023). Early VAP was diagnosed in 15(51.7 %) patients in the selenium group, whereas it was diagnosed in 15 (60 %) patients in the control group (*p* = 0.54). On the other hand, late VAP was diagnosed in 5 (17.2 %) patients in the selenium group and 11 (44 %) in the control group (*p* = 0.032) as described in Table 3.

**Table 3:** Clinical outcomes of study patients

Outcome (s)	Selenium group (*n* = 29)	Control group (*n* = 25)	*P* value
SOFA respiratory score			
Day 0	2.3 ± 0.8	2.4 ± 0.8	0.82
Day 3	2.5 ± 0.9	2.4 ± 0.9	0.69
Day 7	2.1 ± 1	2.7 ± 1	0.03
Day 10	1.6 ± 1.2	2.5 ± 0.8	0.01
Day 14	2.2 ± 1.4	2.4 ± 0.9	0.77
VAP,* n* (%)	16 (55.2)	21 (84)	0.023
Early VAP, *n* (%)	15 (51.7)	15 (60)	0.54
Late VAP, *n* (%)	5 (17.2)	11 (44)	0.032
Duration of mechanical ventilation (in days), mean ± SD	8.9 ± 5	10.7 ± 4.5	0.17
Length of ICU stay (in days), mean ± SD	19.7 ± 11	23.8 ± 13	0.37
Lengths of hospital stay (in days), mean ± SD	25.2 ± 10	24.5 ± 9	0.87
28-day mortality, *n* (%)	9 (31)	10 (40)	0.49

*SOFA* sequential organ failure assessment, *EVAP* early ventilator-associated pneumonia, *LVAP* late ventilator-associated pneumonia, *ICU* intensive care unit

*Effect of VAP on clinical outcomes* We also investigated the effect of VAP on the measures of health care resource consumptions such as duration of mechanical ventilation, duration of ICU and hospital stay and days on vasopressor support. Our results showed that there was no significant difference between those with early VAP versus no early VAP in terms of these measures. However, the presence of late VAP significantly increased the duration of mechanical ventilation (13.3 ± 1.4 versus 8.2 ± 4.8, *p* < 0.0001), duration of ICU stay (33 ± 17 versus 16.5 ± 13, *p* < 0.0001), duration of hospital stay (37 ± 22 versus 19.3 ± 15.6, *p* = 0.002) and days on vasopressor therapy (8.3 ± 5.3 versus 2.4 ± 3, *p* < 0.0001). Similarly, early VAP had no significant effect on the incidence of reinfection (*p* = 0.60), ARDS (*p* = 0.47) and overall mortality (*p* = 0.54). On the other hand, there was significant difference between patients presented with late VAP versus no late VAP with respect to the incidence of new infection (OR 46; 95 % CI 8–259, *p* < 0.0001) and overall mortality (OR 10; 95 % CI 0.18–47, *p* = 0.001), without statistically significant effect on the incidence of ARDS (*p* = 0.087) as depicted in Table 4.

**Table 4:** The effect of VAP on clinical outcomes in study patients

Outcome (s)	Early VAP		OR [95 % CI]	*P*	Late VAP		OR [95 % CI]	*P* value
	Yes	No			Yes	No		
DOMV, mean ± SD	10.6 ± 4	8.6 ± 5.5		0.17	13.3 ± 1.4	8.2 ± 4.8		<0.0001
Duration of ICU stay, mean ± SD	22.9 ± 15.5	20 ± 17.9		0.54	33 ± 17	16.5 ± 13.4		<0.0001
Duration of hospital stay, mean ± SD	25.5 ± 17	24 ± 23		0.78	37 ± 22	19.3 ± 15.6		0.002
Days on vasopressor therapy, mean ± SD	3.4 ± 4	5.5 ± 5		0.13	8.3 ± 5.3	2.4 ± 3		<0.0001
Incidence of new infection, *n*/*N*	8/33	8/21	0.6 [0.18–2]	0.60	13/19	3/35	46 [8–259]	<0.0001
Incidence of ARDS, *n*/*N*	3/33	4/21	0.57 [0.11–2.8]	0.47	4/19	3/35	3.9 [0.76–20]	0.087
Overall mortality, *n*/*N*	16/33	13/21	0.97 [0.33–2.8]	0.54	14/19	15/35	10 [1.8–47]	0.001

*DOMV* duration of mechanical ventilation, *ICU* intensive care unit, *VAP* ventilator-associated pneumonia, *ARDS* acute respiratory distress syndrome, *SD* standard deviation, *OR* odds ratio

## Discussion

In this study, 54 patients were randomized to standard therapy plus selenium (29 patients) and standard therapy alone (25 patients) and included in the final analysis despite discontinuation of selenium in patients due to rise in serum creatinine after 2 days of infusion. The baseline characteristics of the patients included were not significantly differing between the two groups at randomization as described in Table 1. The most important striking feature in the current study was the inclusion of young patients compared with previously done similar studies [30–33]. Our hospital is a trauma center level I usually giving services for trauma patients. Most of these patients were admitted to the ICU after car/motorcycle injury and traumatic brain injury (TBI) was the most common one. This group of patients stays in the hospital for longer time and die because of recurrent hospital-acquired infections such as pneumonia and sepsis despite adequate resuscitation and appropriate antibiotic therapy. According to new studies [34–36], early goal-directed therapy versus standard therapy has not changed mortality significantly. That tells us other than fluid and antibiotics, other treatment alternatives may have survival benefits such as high-dose selenium administration.

In this study, we performed a RCT to evaluate the effect of high dose of selenium (2 mg IV bolus during 1 h followed by 1.5 mg continuous infusion for 14 days) on some clinical outcomes. The results of larger, multiple-center trial confirmed the efficacy of high-dose sodium selenite supplementation in patients with severe sepsis and septic shock in terms of 28-day mortality reduction [30], along with other works [37–41]. This could not be confirmed in our study, as the 28-day mortality was similar in both groups. This could be due to the underpowered nature of the current study. However, our result supported other previously done trials [22, 32] and meta-analysis in which selenium supplementation in critically ill patients was not reduced 28-day mortality [24]. The result of our study also showed that administration of selenium resulted in a slight tendency toward decreasing the duration of mechanical ventilation and length of ICU stay with little effect on the length of hospital say. Those who were resuscitated with selenium initially ended up with fewer recurrent pneumonia and sepsis and were weaned quicker due to cardioprotective properties of selenium against reperfusion and reoxygenation which may have led to sepsis-related cardiomyopathy and weaning failure and finally they died as result of sepsis-related complications.

There are growing interests in carrying out researches in critically ill patients that incorporated biomarkers to optimize the treatment effects. The signaling pathways in inflammation and oxidative stress in vivo are quite complex that in sepsis, there is always a cross talk between inflammation, oxidative stress and coagulation [39]. Measuring the effect of therapy on biomarkers of oxidative stress does not necessarily reflect their effect on inflammatory biomarkers. Currently, we have a number of published articles on the effect of selenium on oxidative stress in septic patients, but we rarely find articles on the effect of this drug on inflammatory cytokines in this group of patients. We investigated the effect of high-dose selenium on GPX activity and inflammatory cytokines such as IL-6, IL-8 and IL-10. Accordingly, selenium administration was associated with significant increase in the plasma activity GPX from day 3 onwards up to day 14 as reported by many previous studies [10, 22, 30, 31] without no effect on the average plasma levels of the after mentioned three cytokines level at any point in time. Although we aimed to evaluate the effect of selenium on the inflammatory response, the study did not provide any new convincing insights into the mechanisms which have already been investigated in different clinical settings [42]. A more productive line of reasoning is as follows: lack of efficacy on cytokines could be due to the small sample size of the study. Second, it seems that monovalent approaches in isolation are unlikely to attain status of complete therapy due to complex interplay between different inflammatory pathways. Saying in another way, it seems that the production and release of sepsis mediators should be considered as a network rather than as a cascade; consequently, once the process is started, even if one of the substances responsible for the initial phase is blocked, other mediators will likely maintain the septic response [43, 44]. Therefore, as sepsis is such a complex disease process, it is unlike that any single agent can be effective for all patients. A combination strategies—the so-called ‘’cocktail’’—can more likely produce definitive results. This can be supported by previous study involving 165 patients requiring mechanical ventilation for sepsis or septic shock. According to this study, patients were randomized to receive enteral nutrition with a standard formula or a study formula that contained higher levels of omega-3 fatty acids and γ-linolenic oil, less omega-6 fatty acids, and higher doses of the antioxidant vitamins E and C and selenium. Patients receiving the study formula had an improvement in their mortality rate, oxygenation, ventilator-free days, ICU-free days, and less development of new organ dysfunction [45].

Moreover, the short-half lives and blood levels of cytokines are variable, transit and non-specific so that their measurement might not be viable and effective way of monitoring the efficacy of therapeutic agent. Study by Landenberg G et al. on myocardial dysfunction in 105 severe sepsis and septic shock patients by repeated echocardiograms and concurrent serum inflammatory cytokines (IL-1β, IL-6, IL-8, IL-10, IL-18, tumor necrosis factor-α, and monocyte chemoattractant protein-1) and cardiac biomarkers [high-sensitivity troponin-T and N-terminal pro-B-type natriuretic peptide (NT-proBNP)] measuring showed that none of the measured inflammatory cytokines correlates with systolic or diastolic myocardial dysfunction in this group of patients. The implication of this study is that there may be no correlation between inflammatory cytokines in real-life clinical setting with clinical severity of the diseases. Therefore, the missing effect of selenium on cytokines under consideration does not mean that selenium has no role in septic patients [46]. There is also a hypothesis that differentiation of CD4 helper cells into TH1 and TH2 is dependent on the delicate amount of selenium available. However, Selenium supplementation boosts T cell receptor signals and skews differentiation toward a Th1 phenotype and Selenium deficiency leads to low T cell receptors signals and skews differentiation toward lowered activation states with a bias toward a Th2 phenotype; adequate selenium intake does not bias T cell differentiation so that the cytokines released are balanced, which could be the reason why we could not see the effect of selenium in this study [42].

The mean SOFA respiratory scores, which assess the degree of respiratory dysfunction decreased significantly in selenium group, compared with the control on days 7 and 10. This indicates that selenium supplementation in critically ill patients reduces pulmonary infections and thereby improves pulmonary function. A study showed that in patients with severe burn trauma, an adjuvant selenium substitution reduced mainly pulmonary infections [47]. Moreover, the administration of high-dose selenium was associated with significant reduction of occurrence of VAP. Although the occurrence of late VAP was significantly reduced, there was no significant difference with regard to occurrence of early VAP between the two groups; a result opposite with that of Manzanares et al. [33],where selenium supplementation in critically ill patients with SIRS associated with significant reduction in early VAP. The explanation for the discrepancy is that peripheral tissues represent a large and slowly recycling pool. The tissue selenium concentration increases at a lower rate to the normal level compared with the plasma concentration and it begins to increase after selenium supplementation for 2 days and then continues to increase slowly for several weeks [43].Therefore, it makes sense that selenium supplementation slowly increases the intracellular concentration of selenium and the activity of glutathione peroxidase-3, an enzyme that prevents the damaging of endothelial cells and the adherence of bacteria into the respiratory mucosal cells and thereby infection. From this perspective, it is logical if selenium supplementation prevents late-onset VAP which happens in patients after 5 or more days on mechanical ventilation. Nevertheless, it is conceivable from the two studies that the contribution of VAP to mortality and ICU resource consumption makes prevention an attractive approach, one of which selenium is proposed as novel strategy for inclusion into the bundles for VAP prevention [29]. The damaging of pulmonary interstitial and alveolar spaces in the lung begins with the interaction of immune cells with the endothelial cells, which stimulates the later to express surface adhesion molecules that bind to the neutrophils and facilitate their migration into the interstitial and alveolar spaces. There is also concomitant triggering of free radical bursts into the lungs [33]. Selenium being antioxidant and anti-inflammatory plays a great role here by increasing the activity of glutathione peroxidase. Unfortunately, suboptimal immune function in patients with selenium deficiency was observed, whereby the functions of both innate and acquired immune system impaired and leading to infection, multiple organs failure and hospital-acquired pneumonia [21]. Our study similarly showed that selenium supplementation reduced VAP and reinfection as well. There are also a couple of supporting ideas from Scottish Intensive Care Glutamine or Selenium Evaluative Trial (SIGNET) [23] and Berger et al. [47, 48] that enhancing trace elements in critically ill patients is associated with decreased hospital-acquired infections such as episodes of VAP.

Our analyses included versatile directions in that we also did analysis of the effect of VAP on overall mortality, the incidences of reinfection and ARDS and health care resource consumption measures such as duration of mechanical ventilation, duration of ICU and hospital stay, days on vasopressor therapy to explore the indirect beneficial effects of selenium on these clinical outcomes. Accordingly, we found that patients presented with late VAP had significantly longer duration of mechanical ventilation, ICU stay, hospital stay and vasopressor therapy compared to those without late VAP. The presence of late VAP was also associated with higher overall mortality, higher incidence of new infection and ARDS, a result in line with some previously done studies [13, 49–52]. On the other hand, there was no significant difference in terms of the mortality, incidence of reinfection or ADRS and resource consumption measures in patients with early VAP compared to those with no early VAP.

Our study has a couple of strengths. First, we administered selenium within the time frame of early goal-directed therapy to evaluate its effect early in sepsis, a strategy found to reduce the mortality rate significantly in patients with severe sepsis and septic shock [53, 54], although this issue becomes a controversial issue at this moment [34–36]. Second, we investigated the effects of VAP on different outcomes on mechanically ventilated septic patients for 5 or more days, which is very important for clinicians caring for such patients to take action ahead of the occurrence and the hospital as a whole to reduce the resource consumption. However, our study was not without limitations. Interpretation of the result of our study should be in caution since our sample size was underpowered to detect the expected differences between the two groups in terms of outcomes. It is also important to mind that our study was single centered which did not include patients from different geographical areas, ethnicity and limited number of females and therefore it perhaps lacked external validity. Furthermore, the study period was relatively long to enroll the required number of participants. Unfortunately, the number of participants enrolled was much below our plan since we are caring for much debilitated patients referred from other centers around that require intensive monitoring and our restricted inclusion criteria. Finally, it is perceivable that the current study was single blind study which could possibly introduce bias to the outcomes.

## Conclusion

High-dose selenium administration within the time frame of early goal-directed therapy was not resulted in reduction of 28-day mortality, but increased the activity of glutathione peroxidase with no effect on the levels of inflammatory cytokines at any point in time in mechanically ventilated septic patients. However, selenium supplementation in mechanically ventilated patients following sepsis was associated with reduced occurrence of VAP. We also observed that late-onset VAP has a significant effect on clinical outcomes and health care resource consumption measures. It is prudent to evaluate the result of this study by an adequately powered, randomized, placebo-controlled trial of high methodological value.
